# Patterns of Detoxification Enzyme Activities During the Selection of *Phortica okadai* Resistant to β-Cypermethrin Under Laboratory Conditions

**DOI:** 10.3390/insects16040346

**Published:** 2025-03-26

**Authors:** Juan Zhou, Fang Wu, Yang Luo, Zhenfu Chen, Donghua Long, Hui Liu, Bo Luo, Rong Yan, Lingjun Wang

**Affiliations:** 1 Department of Parasitology, Zunyi Medical University, Zunyi 563000, China; 2 Department of Pathological Biology and Immunology, Zunyi Medical and Pharmaceutical College, Zunyi 563000, China

**Keywords:** *Phortica okadai*, β-cypermethrin, resistance selection, detoxification enzyme

## Abstract

After eight generations of laboratory selection with β-cypermethrin, a *P. okadai* strain was cultivated that displayed a 10.04-fold increase in resistance (RS) relative to the susceptible strain (SS), with an estimation of heritability (*h*^2^) of 0.34. Compared with the SS, the developmental duration of the eggs was significantly prolonged (*p* < 0.05); however, the pupal stage duration was shorter, with no statistically significant difference. Moreover, the levels of GSTs, CarEs, and CYP450 activity were notably higher in the RS than in the SS. In addition, the level of CarE and CYP450 activity in the RS was significantly higher in the midgut (MG), fat body (FB), and Malpighian tubules (MTs) compared to the SS; however, the GSTs showed no statistically significant difference in the MTs. These results suggest that *P. okadai*‘s resistance to β-cypermethrin could be selected rapidly and the decreases in the intrinsic rate of increase (r) observed in the RS are likely due to mutations in the detoxification enzyme genes under the strong selection pressure exerted by β-cypermethrin. The increased activity of GSTs, CarEs, and CYP450 was associated with β-cypermethrin resistance in the RS of *P. okadai.* The data reported herein provide a foundation for future studies on the mechanisms responsible for β-cypermethrin resistance in *P. okadai.*

## Simple Summary

In China, *Phortica okadai* is the only established vector of *Thelazia callipaeda*. β-cypermethrin is a pesticide commonly used for the control of agricultural pests that may pose a risk to *P. okadai* due to the potential development of pesticide resistance. A strain of *P. okadai* moderately resistant to β-cypermethrin (RS) was obtained after laboratory selection with a 10.04-fold increase in resistance (vs. the susceptible strain, SS). We compared the development duration and life table of the RS and SS, and the activities of carboxylesterases (CarEs), cytochrome P450 (CYP450), and glutathione S-transferases (GSTs) in different development stages and tissues of *P. okadai* in the RS and SS were measured. The results presented in this study provide critical baseline data for monitoring resistance evolution and optimizing vector control strategies against *P. okadai*.

## 1. Introduction

In nature, *Phortica okadai* (Máca) (Drosophilidae: Steganinae) comes into contact with *Thelazia callipaeda* (Railliet and Henry) (Spirurida: Thelaziidae) and acts as its intermediate host. It transmits the infective larvae of *Thelazia callipaeda* to various animals, including wild and domestic lagomorphs and carnivores, and humans. *Phortica okadai* is the only established vector of *T. callipaeda* in China [1,2,3,4].

Insecticides play a central role in controlling major vectors of disease to reduce the transmission of vector-borne infectious diseases. Cypermethrin is one of the most widely used synthetic insecticides for agricultural and domestic purposes. Its use, however, has led to resistance in the target insect vectors to insecticides [5], representing a major issue in pest control. Moreover, it has also been identified as one of the leading constituent pesticides associated with risks to human health [6]. Resistance to cypermethrin is reported in many insect pests such as *Drosophila melanogaster* (Meigen) and *Spodoptera littoralis* (Boisduval) [7,8]. Under long-term insecticide selection pressure, insects develop resistance to protect themselves from the damage caused by insecticides. Understanding the response of detoxification factors to insecticide stress may help in elucidating the mechanisms of insecticide-induced tolerance.

Research has confirmed that insects gain resistance to insecticides using a variety of methods, often using more than one mechanism simultaneously, such as decreased penetration, altered target site insensitivity, and metabolic resistance mechanisms mediated by detoxifying enzymes [9]. Metabolic resistance involving an array of detoxification enzymes, such as cytochrome P450 monooxygenase, esterase, and glutathione S-transferase, is the primary mode of insecticide detoxification [10,11,12]. In-depth studies on the mechanisms of insect metabolic resistance have shown that insects can achieve enhanced insecticide metabolism by upregulating the transcription of key metabolic enzyme genes, thereby increasing the expression of relevant enzymes [13]. CYP450 can catalyze the metabolism of insecticides and other exogenous harmful substances to reduce the sensitivity of insects to insecticides. A mutation in Cyp6a23 or Cyp6a17 represents a potential source of CYP-mediated resistance in zata-cypermethrin [7,14]. Carboxylesterases (CarEs) can hydrolyze insecticides to enhance insect resistance, and research has shown that the mutation and overexpression of related genes are two primary mechanisms underlying CarE-mediated insecticide resistance, with overexpression of CarE genes being more widely reported [15,16]. For example, overexpression of PxαE8 is responsible for resistance to beta-cypermethrin in *Plutella xylostella* (L.) [17]. GSTs play a crucial role in phase II detoxification metabolism and protection against oxidative stress caused by insecticide exposure. In one study, GstE4 was overtranscribed when *D. melanogaster* was exposed to β-cypermethrin, suggesting that GstE4 may be involved in the early stages of the detoxification or sequestration of β-cypermethrin [18].

Changes in detoxification enzyme activities, mode of inheritance, and realized heritability during the resistance selection of *P. okadai* to β-cypermethrin were evaluated in the present study to aid in the development of an improved resistance management strategy. The aim of this study was to provide a basis for effectively controlling the transmission of *T. callipaeda* from the perspective of vector control and present baseline data for insecticide resistance levels of *P. okadai* to β-cypermethrin.

## 2. Materials and Methods

### 2.1. Insect Rearing

Susceptible strain (SS): *P. okadai* were collected from a field in Zunyi, Guizhou Province, China, and were reared in our laboratory without exposure to any insecticides. The insects were maintained in a 30 cm cuboid insect rearing cage and supplied with naturally fermented fruits for 3 days at a temperature of 28 ± 1 °C, 75 ± 5% relative humidity, and a 16L:8D photoperiod.

Resistant strain (RS): Each generation of adults of the susceptible strain *P. okadai* were maintained in the laboratory until selection in November 2021 using the medical membrane method with β-cypermethrin for eight generations.

### 2.2. Reagents

Acetone was purchased from Chengdu Jinshan Chemical Co., Ltd. (Chengdu, China); 96% β-cypermethrin was purchased from Gongcheng Bio-tech Co., Ltd. (Nantong, China); phosphate-buffered saline (PBS) was purchased from Beijing Solaibao Technology Co., Ltd. (Beijing, China); the Bicinchoninic Acid Kit for Protein Determination was purchased from Nanjing Jiancheng Bioengineering Institute (Nanjing, China); and ELISA kits for insect cytochrome P450 (CYP450), carboxylesterases (CarEs), and glutathione S-transferases (GSTs) were all purchased from Jiangsu Meimian Industrial Co., Ltd. (Yancheng, China).

### 2.3. Determination of Sublethal Concentrations and Tolerance Bioassay

The toxicities of adult *P. okadai* to β-cypermethrin were evaluated separately using the medical membrane method following the test methods for fly resistance to insecticides and the bioassay methods for *musca domestica* [19].

Serial dilutions of technical-grade β-cypermethrin (20–80%) were prepared in acetone and for each concentration, three repetitions with 20 adults were carried out. The adult was transferred into a 100 mL serum bottle with 0.5 mL uniformly distributed and volatilized insecticide, with the bottle then covered with a muslin cloth to prevent escape. We established one blank group without reagents and acetone was applied to the control group. Mortality was assessed 3 h after application, determined by the inability to turn over, motionless, and a lack of coordination when attempting such movement. The insects were maintained at a temperature of 28 ± 1 °C and 75 ± 5% relative humidity throughout the experiment.

### 2.4. Resistance Selection

To select the strain resistant to β-cypermethrin under laboratory conditions, the (1-day-old adult) *P. okadai* that had emerged from a relatively susceptible strain was chosen as the F_0_ generation. *P. okadai* were treated using the medical membrane method described by Abbas and Hafez et al. [20] with β-cypermethrin applied every 2–3 days in each generation. The concentration was maintained at the previous generation’s LC_50_. A medical air-compressed nebulizer (Yuyue 405E) was used, with a power of 0.2 mL/min and a pressure range of 70–106 kPa. Air was continuously applied for 2–3 min. After treatment, the surviving individuals were reared further and allowed to reproduce. Once the next generation was produced, the above treatment protocol was repeated. More than 1000 *P. okadai* were treated per generation.

Every two generations, a toxicity test was carried out to calculate the resistance ratio (RR). The resistance ratio was calculated as follows: RR = LC_50_ of the RS/LC_50_ of the SS. The resistance levels were classified using the guideline for insecticide resistance monitoring of the striped stemborer bioassay method for *musca domestica* [21]: no resistance (RR < 5), low resistance (5 < RR ≤ 10), moderate resistance (10 < RR ≤ 100), or high resistance (RR > 100).

### 2.5. Estimation of Realized Heritability (h^2^)

The *h*^2^ values for β-cypermethrin resistance were assessed using the equations of Falconer [22] and Tabashnik [23], which were estimated as follows:

$$h^{2}=\frac{R}{S}$$

In the above equation, *R* (selection response) was estimated as follows:

$$R=\frac{\log_{\mathrm{final}}{LC}_{50}-\log_{\mathrm{initial}}{LC}_{50}}{N}$$

The final LC_50_ is the LC_50_ of the population after two selected generations, the initial LC_50_ is the LC_50_ of the susceptible strain before selection, and N is the number of generations selected with β-cypermethrin, where S (selection differential) was calculated as follows:

*S* = *ixσp*

where *i* is the selection intensity (mortality), determined following the method of Tabashnik and McGaughey [24]

*i* = 1.583 − 0.0193336*p* + 0.0000428*p*2 + 3.65194/*p*

where *i* is the intensity of the selection calculated and *σp* is the phenotypic standard deviation calculated as follows:

$$\sigma p=\frac{1}{mean\;Slope}$$

The number of generations (G) required to produce a ten-fold increase in the median lethal concentration of β-cypermethrin was determined as follows:

$$G_{X}=\frac{{log}_{X}}{h^{2}S}$$

### 2.6. Comparison of the Development Duration and Life Table Between the RS and the SS

A total of 460 *P. okadai* eggs were used, with 230 eggs from each of the RS and SS. Each individual *P. okadai* egg was placed and numbered in a 30 mm Petri dish with the lid replaced with an organdy mesh for ventilation. A piece of pear that had been fermented for 3 days was supplied as food and replaced every other day. The individuals were checked and recorded every 24 h to determine the developmental parameters, including degree-days estimated for egg development (time from oviposition to hatching) and for larval development (larvae to pupae). Once the larvae developed into pupae, they were transferred into a cylindrical plastic box with a base radius of 10 cm and a height of 15 cm covered with wet cotton. The time of eclosion and the number of individuals emerging were observed and recorded every 24 h. After eclosion, the number of emerged adults and their sex were recorded on a daily basis. Male and female adults that emerged on the same day were randomly paired and placed in separate containers sealed with gauze. Each pair was provided with water and fermented pears. During the reproductive period, the newborn eggs were counted and then removed using a soft brush every 24 h until the adult died. The rearing conditions for all the test insects were as follows: temperature, 28 ± 2 °C; relative humidity, 75 ± 5%; and light, 8 h/d.

### 2.7. Statistical Analysis

The data were tested for normal distributions and subsequently analyzed for two-group comparisons (Pearson’s chi-squared test or Student’s *t*-test) using SPSS 16.0 or GraphPad prism 9.0 to compare the differences between the RS and SS. Differences were considered significant at the *p* < 0.05 level.

The life history raw data of individual *P. okadai* were analyzed by using the TWO-SEX-MS Chart program [25] based on the age-stage, two-sex life table theory [26], and the method described by Chi [27]. The survival rate (*s_xj_*) (*x* = age; *j* = stage), which is the probability that a newly laid egg will survive to age *x* and stage *j*, and fecundity *f_xj_*, which is the number of hatched eggs produced by the female adult at age *x*, were calculated. The means and standard errors of developmental duration, in addition to the fecundity and population parameters, were analyzed using bootstrapping methods in the TWOSEX-MS Chart program with 10,000 resamples. All of the data are shown as the mean ± SE.

The age-specific survival rate (*lx*) was then calculated as follows:

$$l_{x}=\sum_{j=1}^{m}s_{xj}$$

where *m* is the number of stages. Age-specific fecundity (*m_x_*) was calculated as follows:

$$m_{x}=\frac{\sum_{j=1}^{m}s_{xj}f_{xj}}{\sum_{j=1}^{m}s_{xj}}$$

The net reproductive rate is defined as the total number of offspring that an individual can produce during its lifetime and is calculated as follows:

$$R_{0}=\sum_{x=0}^{\infty }l_{x}m_{x}$$

The intrinsic rate of increase was calculated using the equation with age indexed from zero and is calculated as follows:

$$\sum_{x=1}^{\infty }(e^{-rx}\sum_{j=1}^{m}f_{xj}S_{xj})=1$$

The mean generation time represents the period that a population requires to increase to *R*_0_-fold of its size as time approaches infinity and the population settles down to a stable age-stage distribution. The mean generation time is calculated as follows:

$$T=\frac{\ln R_{0}}{r}$$

The finite rate of increase indicates the number of times the population multiplies in a unit of time and is calculated as follows:

$$\lambda =e^{r}$$

The mean generation time is calculated as the age-stage-specific life expectancy (*e_xy_*), that is, the time that an individual of age *x* and stage *y* is expected to live, and was calculated according to the method described by Chi and Su [28] as follows:

$$e_{xj}=\sum_{i=x}^{\infty }\sum_{y=i}^{k}s_{iy}^{\prime }$$

where *s*′*_iy_* represents the probability that individuals of age = *x* and stage = *j* will survive to age = *i* and stage = *y*.

The reproductive value is defined as the contribution of individuals of age *x* and stage to the future population. In the age-stage, two-sex life table [28], it is calculated as follows:

$$v_{xj}=\frac{e^{r(x+1)}}{s_{xj}}\sum_{i=x}^{\infty }e^{-r(i+1)}\sum_{y=i}^{k}s_{iy}^{\prime }f_{iy}$$

### 2.8. Determination of Detoxification Enzyme Activities

ELISA kits were used to measure the activity of detoxification enzymes in *P. okadai*. Following the method employed by Cao [29], each RS and SS of 15 larvae (3 days old), 10 pupae (5 days old), and 8 adults (4 days old) of *P. okadai* were used for the different stage enzyme preparation and 50 midgut, 50 fat body, and 60 Malpighian tubules of *P. okadai* (3–5 days old) adults were used for different tissue enzyme preparation. Three replicates were run at each stage and for each tissue. The *P. okadai* were individually homogenized with phosphate-buffered saline (1 g: 9 mL). After being centrifuged at 13,800 rpm for 15 min, the clear supernatant was collected and used as an enzyme resource for analysis of the activities of CYP450, GSTs, and CarEs. All procedures were carried out on ice to minimize the loss of enzyme activity.

The protein content of the enzyme solution was determined by employing the bicinchoninic acid (BCA) method in accordance with the kit’s instructions. CYP450, GST, and CarE activities were determined using ELISA kits following the manufacturer’s protocol. The data obtained from the enzyme assays were subjected to analysis of variance followed by Bonferroni multiple comparison post hoc tests using GraphPad prism 9.0.

## 3. Results

### 3.1. Estimation of Realized Heritability and Resistance Rate

After eight generations of selection with β-cypermethrin in the laboratory, the resistance ratio (RR) rose from 1.000 at the F_0_ generation to 10.04 at the F_8_ generation. Based on the test methods of fly resistance to insecticides and the bioassay methods for *musca domestica* [19], *P. okadai* gained moderate resistance to β-cypermethrin (Table 1).

The LC_50_ value of *P. okadai* increased from 0.408 to 1.667. The value calculated for the estimation of realized heritability (*h*^2^) was 0.34 (Table 2). This value suggests that resistance to β-cypermethrin had increased across generations under pressure selection in laboratory conditions.

Initial LC_50_ = the LC_50_ of the population F_0_ before selection; final LC_50_ = the LC_50_ of the population after eight selected generations; *R* = log (final LC_50_) − log (initial LC_50_)/n; *p* = percentage of the population with values above the selection threshold; *i* = intensity of selection calculated; *σp* = 1/mean slope, *S* = *iσp*, *h*^2^ = *R*/*S*. G = number of generations screened with β-cypermethrin [20].

**Figure 1:**
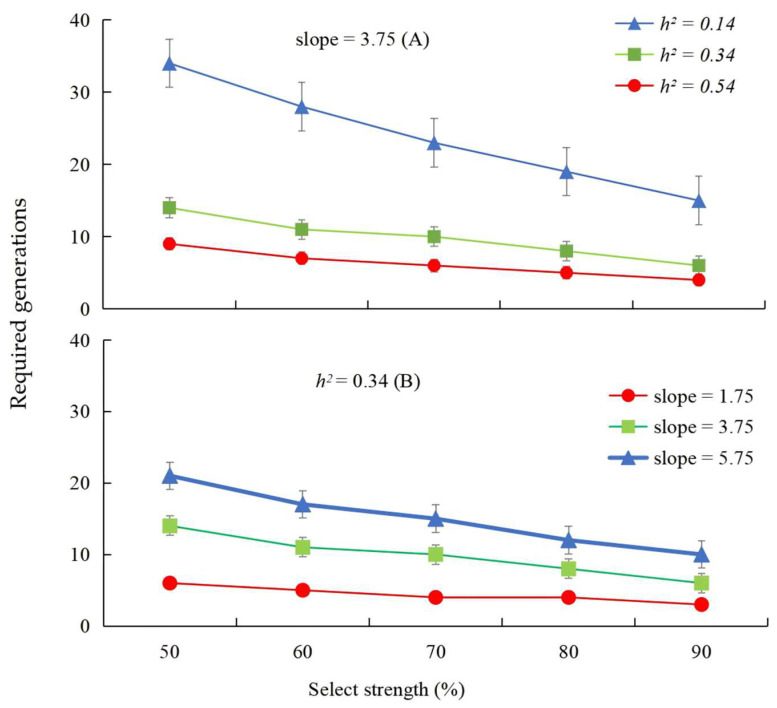
Effects of different realized heritability *h*^2^ (**A**) and slope (**B**) on the number of generations required for a ten-fold increase in the LC_50_ of β-cypermethrin at different selection intensities.

At different *h*^2^ values of 0.14, 0.34, and 0.54 with a constant slope value of 3.75, 15–34, 6–14, and 4–9 generations would be needed to induce a ten-fold increase in the LC_50_ of β-cypermethrin in the resistant strain of *P. okadai*, causing 50–90% mortality (Figure 1A).

At an *h*^2^ value of 0.34 and with slope values of 1.75, 3.75, and 5.75, 6–3, 14–6, and 21–10 generations would be needed to induce a ten-fold increase in the LC_50_ of β-cypermethrin (Figure 1B). These results suggest that the risk of developing resistance to β-cypermethrin may increase to high levels if the selection pressure is continued intensively.

### 3.2. Comparative Development Duration and Life Table Between the RS and SS

There was a significant difference between the RS and SS of *P. okadai* in the duration of the egg, larval, and pupal stages, with durations of 1.90 ± 0.04 and 1.43 ± 0.05 days in the egg stage, 6.26 ± 0.13 and 5.62 ± 0.09 days in the larval stage, and 9.27 ± 0.14 and 10.84 ± 0.1 days in the pupal stage. No statistically significant difference was observed in the adult pre-ovipositional periods (APOPs) and the total pre-ovipositional periods (TPOPs), with durations of 11.12 ± 0.78 and 9.74 ± 0.35 days in the RS and 27.68 ± 0.89 and 26.96 ± 0.41 days in the SS. The development duration of the RS was longer than that of the SS in the egg and larval stages, APOPs, and TPOPs but shorter than that in the pupae (Table 3). 

Table 4 presents the age-stage, two-sex life table parameters of the two strains of *P. okadai*. The intrinsic rate (*r*), finite rate of increase (*λ*), and the net reproductive rate (*R*_0_) showed significant differences among the RS and SS. The *r*, *λ*, and *R*_0_ were 0.0448 ± 0.0093 day^−1^, 1.0458 ± 0.0097 day^−1^, and 6.3260 ± 2.1712 in the RS offspring, and 0.090 ± 0.0039 day^−1^, 1.0943 ± 0.0042 day^−1^, and 32.1565 ± 4.1889 in the offspring of the SS. No statistically significant difference was observed in the mean generation time (*T*) among the RS and SS, with durations of 41.205 ± 1.7490 days for the RS and 38.5930 ± 0.5900 days for the SS.

**Figure 2:**
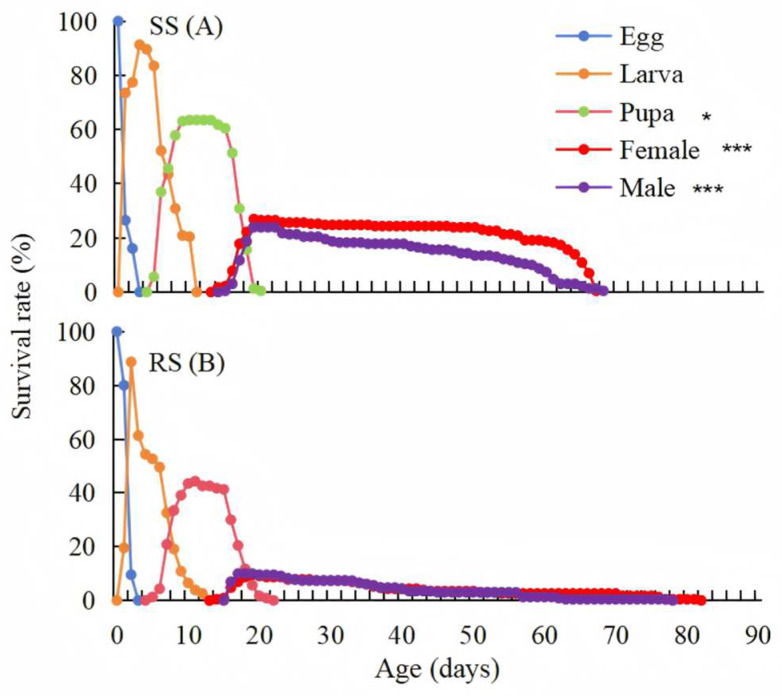
Age-stage-specific survival rates (*s_xj_*) of the SS (**A**) and RS (**B**) of *P. okadai.* ***: *p* < 0.001; *: *p* < 0.05.

The age-stage-specific survival rate (*s_xj_*) curves indicate the probability that an egg of *P. okadai* will survive to age *x* and develop to stage *j.* There was a significant difference of *s_xj_* between the RS and SS of *P. okadai*, with survival rate of 63.48 and 44.35% in the pupal (t = −2.227, df = 33, *p* = 0.033, ci = −0.318, −0.143), 26.96, and 9.13% in the female (t = −7.379, df = 134, *p* < 0.001, ci = −0.163, −0.094), 23.91, and 9.57% in the male (t = −9.94, df = 116, *p* < 0.001, ci = −0.117, −0.784) (Figure 2).

**Figure 3:**
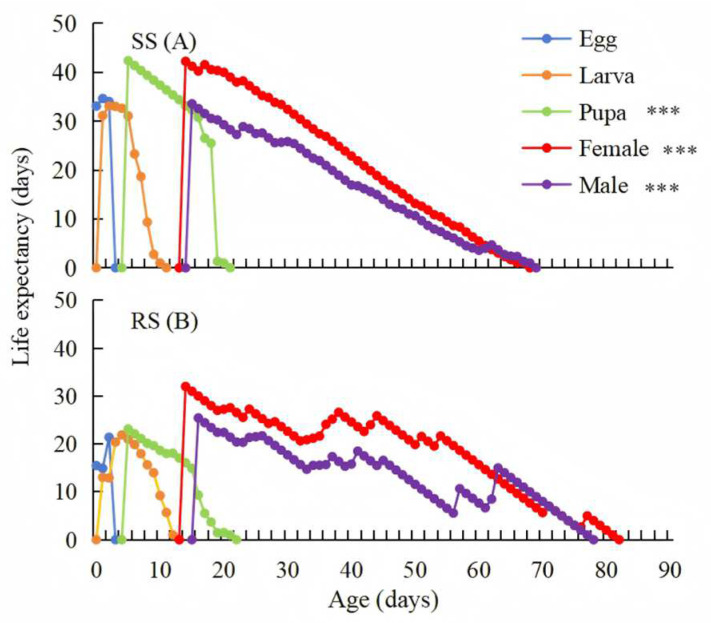
Age-stage-specific life expectancy (*e_xj_*) of the SS (**A**) and RS (**B**) of *P. okadai.* ***: *p* < 0.001.

The age-stage-specific life expectancy (*e_xy_*) is defined as the length of duration that an individual of age *x* and stage *y* is expected to live after age *x*. There was a significant difference of *e_xj_* between the RS and SS of *P. okadai*, with life expectancy of 23.14 and 42.38 days in the pupal (t = −3.764, df = 35, *p* < 0.001, ci = −23.702, −7.042), 32.00 and 42.45 days in the female (t = −3.382, df = 220, *p* < 0.001, ci = −8.344, −2.199), 25.43 and 33.57 days in the male (t = −3.333, df = 214, *p* < 0.001, ci = −6.192, −1.59) (Figure 3).

**Figure 4:**
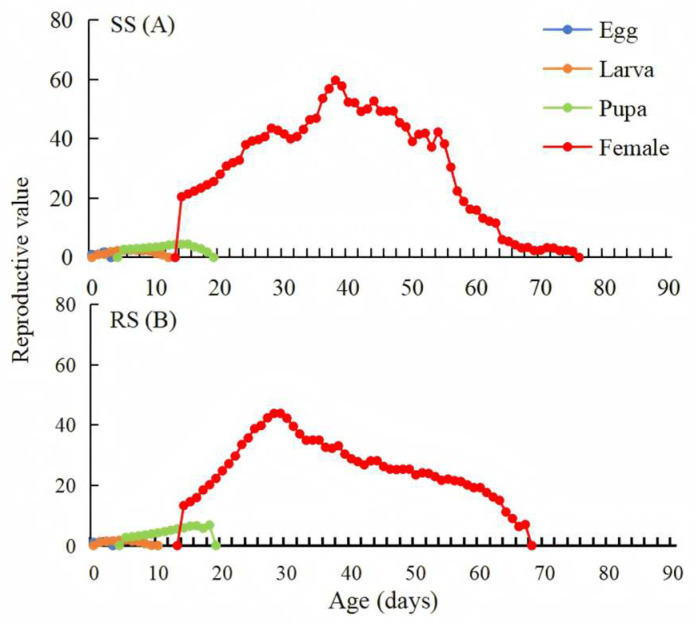
Age-stage-specific reproductive value (*v_xj_*) of the SS (**A**) and RS (**B**) of *P. okadai*.

The age-stage-specific reproductive values (*v_xj_*) of *P. okadai* represent the contribution of an individual at age *x* and stage *j* to the future population. An increase with no significant difference in *v_xj_* was observed at 13 days of age in both the RS and SS. The RS maintained a high rate of reproduction at 38 days of age; in comparison, the SS maintained a high reproduction rate at 29 days of age (Figure 4).

### 3.3. Effect on Detoxification Enzyme Activities

**Figure 5:**
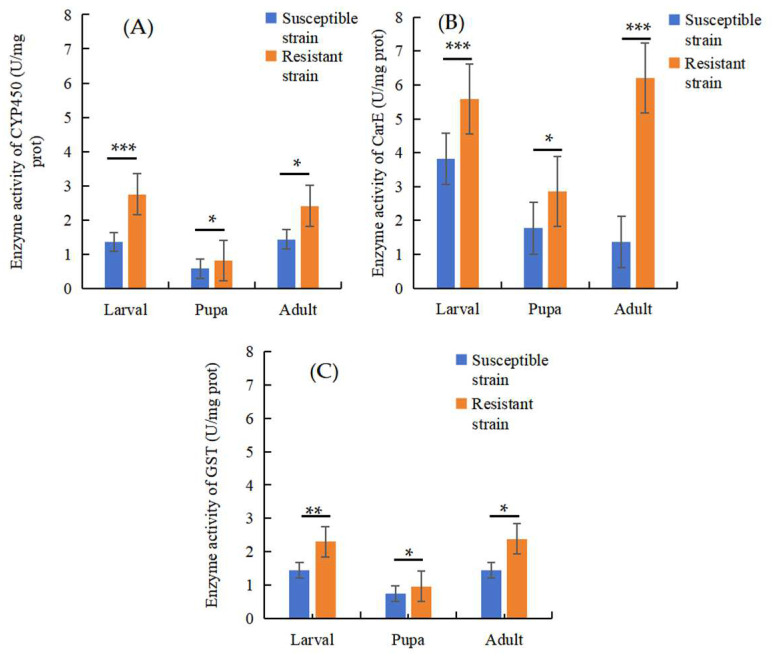
Changes in CYP450 (**A**), CarE (**B**), and GST (**C**) activity in the RS and SS at different stages of development. ***: *p* < 0.001; **: *p* < 0.01; *: *p* < 0.05.

To determine the role of detoxification enzymes in the resistance of *P. okadai* to the pesticide β-cypermethrin, detoxification enzyme assays were conducted to measure the activities of CarEs, GSTs, and CYP450. The results showed that there was a statistically significant difference in the enzyme activities of CarEs (larvae: t = 10.18, df = 4, *p* = 0.0005, ci = 1.280–2.240; pupae: t = 4.189, df = 4, *p* = 0.0138, ci = 0.3665–1.807; adult: t = 8.873, df = 4, *p* = 0.0009, ci = 3.315–6.334), GSTs (larvae: t = 5.708, df = 4, *p* = 0.0047, ci = 0.4416–1.278; pupae: t = 2.934, df = 4, *p* = 0.0426, ci = 0.01200–0.4351; adult: t = 3.888, df = 4, *p* = 0.0177, ci = 0.2700–1.619), and CYP450 (larvae: t = 8.64, df = 4, *p* = 0.001, ci = 0.9440–1.838; pupae: t = 3.728, df = 4, *p* = 0.02, ci = 0.0613–0.4192; adult: t = 2.96, df = 4, *p* = 0.04, ci = 0.0604–1.885) between the RS and SS in the larvae, pupae, and adults. The highest level of activity of the CarEs and GSTs was observed in the adults; however, the highest level of CYP450 activity was observed in the larvae (Figure 5).

**Figure 6:**
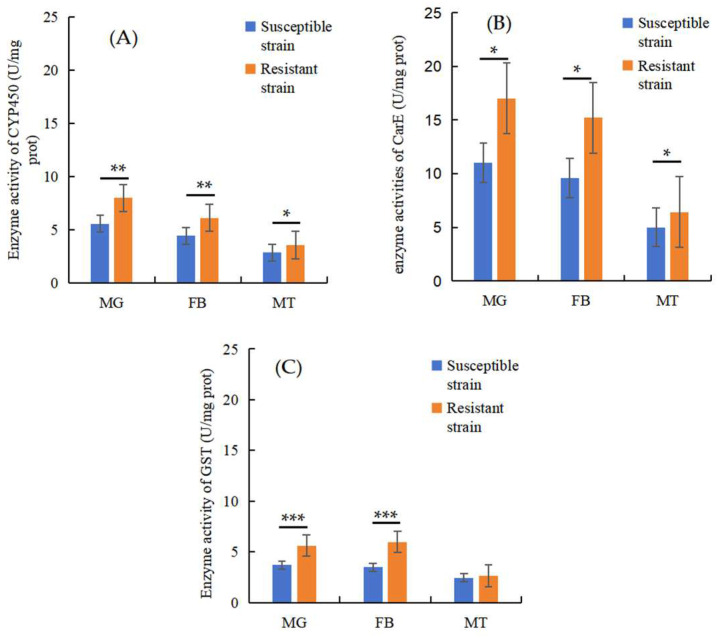
Changes in CYP450 (**A**), CarE (**B**), and GST (**C**) activity in the RS and SS in different tissues. ***: *p* < 0.001; **: *p* < 0.01; *: *p* < 0.05.

The highest level of CarE and CYP450 activity was observed in the MG; however, the highest level of GST activity was observed in the FB. There was a statistically significant difference in the enzyme activity of CYP450 (MG: t = 6.196, df = 4, *p* = 0.0034, ci = 1.346–3.532; FB: t = 5.108, df = 4, *p* = 0.0069, ci = 0.7808–2.640; MTs: t = 3.684, df = 4, *p* = 0.0211, ci = 0.1781–1.267) and CarEs (MG: t = 4.37, df = 4, *p* = 0.012, ci = 1.769–7.932; FB: t = 4.12, df = 4, *p* = 0.0146, ci = 1.530–7.853; MTs: t = 2.798, df = 4, *p* = 0.0489, ci = 0.0109–2.848) between the RS and SS in the MG, FB, and MTs. The GSTs showed a statistically significant difference in enzyme activity between the RS and SS in the MG (t = 10.2, df = 4, *p* = 0.0005, ci = 1.330–2.324) and FB (t = 9.032, df = 4, *p* = 0.0008, ci = 1.653–3.120) but no significant difference in the MTs (t = 10972, df = 4, *p* = 0.1199, ci = −0.0895–0.5284) (Figure 6).

## 4. Discussion

Insecticides are used to control disease vectors to reduce the transmission of vector-borne infectious diseases. Cypermethrin is a synthetic insecticide widely used for agricultural and domestic purposes; however, resistance due to its overuse has become a major obstacle to effective pest control. Monitoring resistance in *P. okadai* will not only aid in the formulation of suitable insecticides to target this pest but also for the tracking of the precise changes in resistance to β-cypermethrin.

To better understand the mechanism underlying *P. okadai*‘s resistance to β-cypermethrin, a resistant strain was examined in our laboratory. Our results showed that after continuous selection of *P. okadai* from F_1_ to F_8_, the resistance ratio increased to different degrees, with *h*^2^ values of 0.34- and 10.04-fold increase in the resistance strain being obtained after eight generations of selection with β-cypermethrin. Based on realized heritability (*h*^2^) determined through short-term insecticide selection experiments in the laboratory population, we aimed to elucidate the frequency distribution of resistance genes and predict the future development trend of insecticide resistance in these populations [30]. A high *h*^2^ value indicates a higher risk of genetic resistance development, as it suggests that resistance genes are more effectively inherited by subsequent generations, leading to the rapid accumulation of resistance traits within the population. Conversely, a low *h*^2^ value suggests that the observed resistance may be influenced to a greater degree by phenotypic variation, which could result from factors such as gene mutations, population migration, selection pressure, insecticide rotation, and environmental influences, both in laboratory settings and field conditions [31]. These factors introduce additional variability that may not be as strongly genetically based, thus potentially slowing the development of resistance in the population. Estimating the rate of resistance development through the number of generations is a valuable step toward establishing rational resistance management strategies for insect vectors [32]. *M. domestica* exhibited a low probability of developing genetic resistance to alpha-cypermethrin, with an *h*^2^ = 0.14–0.1, 12 generations being required to achieve resistance. In this study, the higher *h*^2^ value of 0.34 (vs. *M. domestica*) suggested that only several generations may be needed for *P. okadai* to reach a significant resistance level compared with *M. domestica*, which provides evidence pertaining to the potential of resistance development. As a result, the application of β-cypermethrin to control *P. okadai* should be approached with caution.

After exposure to insecticides, insects sometimes reduce the level of toxicity to which they are exposed through detoxification enzymes and, simultaneously, changes in development and reproduction to adapt to the environment [33]. Life history traits have been widely used in research to calculate the growth rate influenced by abiotic factors of a resistant population, which is affected by development duration, female or male longevity, larvae fecundity, and viability. In the current study, significant differences were found between the RS and SS of *P. okadai* with prolonged durations of the egg and larval stages. The RS showed no statistically significant difference in prolonging the APOPs and TPOPs but showed a shortened pupal duration compared with the SS. These findings may be the result of the accumulation of sufficient nutrients during the larval stage. A similar trend was found in the β-cypermethrin-resistant population of *Drosophila melanogaster*, in which egg development was inhibited, which may be due to the egg development stage being more sensitive to toxicity than the other stages [20]. The RS exhibited significantly lower values of *r*, *λ*, and *R*_0_ compared with the SS. A significantly decreased *s_xj_*_,_ in the pupal and adult stages of the RS might lead to a significant decrease in *r*, which is highly likely due to tissue damage in reproductive organs and increased stress gene expression (HspVO) under the strong resistance selection pressure induced by β-cypermethrin [34].

Studies in the literature indicate that the enhanced enzymatic detoxification may be related to pesticide resistance, with GSTs, CarEs, and CYP450 being key enzymes in the metabolism of pesticides before they reach the target site [35]. However, the mechanism underlying *P. okadai*’s resistance to β-cypermethrin has not been reported in other studies. In this study, to investigate the correlation between the detoxifying enzyme activities and resistance of *P. okadai* to β-cypermethrin, we compared the activities of constitutive CarE, GST, and CYP450 proteins present in extracts from various stages and tissues of the RS and SS. Our results showed that the activities of GSTs, CarEs, and CYP450 in the RS during each stage were significantly higher than those in the SS, with CYP450 showing the most significant difference in the larval stage and CarEs showing the greatest difference in multiple stages, namely in the pupal and adult stages. The above results may indicate that the elevated levels of GST, CarE, and CYP450 activity contributed to β-cypermethrin resistance in the RS of *P. okadai*. During the larval stage, CYP450 contributed significantly to detoxification; in comparison, during the pupal and adult stages, CarEs primarily play a more important role. Gene amplification and overexpression are two key mechanisms by which insects develop resistance to insecticides. Through gene amplification, the copy number of carboxylesterases (CarEs) in tissues such as the midgut, fat bodies, and Malpighian tubules increases, significantly elevating both the total quantity and enzymatic activity of CarEs. Such changes enhance their capacity to metabolize insecticides. For example, in one study, the mosquito *Culex quinquefasciatus* developed markedly increased resistance to organophosphate through extensive amplification of CarE genes [36]. Overexpression of CarEs at the transcriptional level also plays a critical role in insecticide resistance. This particular mechanism involves transcriptional regulation to boost enzyme activity, leading to the substantial upregulation of CarE gene expression in resistant insect populations. For instance, in one study, multiple overexpressed PxαE6 and PxαE9 genes in *P. xylostella* mediated resistance to beta-Cypermethrin [37]. The fat body (FB), midgut (MG), and Malpighian tubules (MTs) are commonly identified as the main tissues for detoxification [38]. In this study, the activity of CarEs and CYP450 in the RS was significantly higher than that in the SS in the MG, MTs, and FB; however, there was no statistically significant difference in GST activity in the MTs. These results may indicate that the MG, MTs, and FB were the main tissues for detoxifying CarEs and CYP450, with the MG and FB being the main tissues for detoxifying GSTs. Moreover, the MG showed the highest levels of CarEs, GSTs, and CYP450 activity, which may be due to the fact that it is one of the first tissues to come into contact with the chemical when the organism ingests contaminated food [39].

## 5. Conclusions

This study is the first report on resistance selection to β-cypermethrin conducted under laboratory conditions in a susceptible population of *P. okadai*. We found that resistance increased from 1.00- to 10.04-fold after eight consecutive generations of selection compared with the laboratory susceptible strain, thus reflecting the possibility of β-cypermethrin resistance development in this pest species if the insecticide is applied continuously for a long period of time. The RS exhibited a developmental disadvantage compared with the SS, which suggests that resistance to β-cypermethrin in *P. okadai* comes at the cost of prolonged egg and larval development. Additionally, the levels of CarE, GST, and CYP450 activity in the RS *P. okadai* were higher, indicating that they may be involved in the detoxification-based resistance of *P. okadai* to β-cypermethrin. In light of the above results, different insecticides should be selected based on the susceptibility of *P. okadai* populations to insecticides in order to improve control effects. Such data may aid in the development of integrated management strategies for more efficient population control of *P. okadai*.

## Figures and Tables

**Table 1:** Resistance selection of *P. okadai* to β-cypermethrin.

Generations	Slope ± SE	LC_50_ (95% Confidence Interval) (mg/L)	Resistance Ratio (RR)	X^2^ (df)	*p* (95% Confidence Interval)
F_0_	1.496 ± 0.357	0.166 (0.085, 0.228)	1.000	1.143 (3)	0.767 (0.851, 2.988)
F2	2.667 ± 0.281	0.408 (0.383, 0.434)	2.460	0.984 (3)	0.805 (5.224, 7.839)
F4	2.776 ± 0.221	1.450 (1.281, 1.698)	8.730	5.531 (3)	0.137 (1.580, 2.248)
F8	4.659 ± 0.517	1.666 (1.609, 1.727)	10.040	4.023 (3)	0.259 (2.180, 3.414)

LC_50_ = median lethal concentration; resistance ratio = LC_50_ of the RS/LC_50_ of the SS. SE = standard error. Resistance ratio = LC_50_ of the RS/LC_50_ of the SS [20].

**Table 2:** Realized heritability of the resistance strain of *P. okadai* with β-cypermethrin resistance for eight generations.

G	Estimation of Mean Selection Response per Generation			Estimation of Mean Selection Differential per Generation				Estimation of Heritability (*h*^2^)
	Initial LC_50_ (95% Confidence Interval)	Final LC_50_ (95% Confidence Interval)	Response to Selection (*R*)	Intensity of Selection (*i*)	Mean Slope	Phenotypic Variation (*σp*)	Selection Differential (*S*)	
F_0_–F_8_	0.408 (0.383–0.434)	1.667 (1.609–1.727)	0.08	0.85	3.75	0.27	0.23	0.34

**Table 3:** Development difference at each stage of the two strains of *P. okadai*.

Insect Stage	Resistant Strain (Mean ± SEM) (d)	Susceptible Strain (Mean ± SEM) (d)	t (df)	*p* (95% Confidence Interval)
Duration in the egg stage	1.90 ± 0.04 a	1.43 ± 0.05 b	7.766 (457)	<0.001 (0.354, 0.593)
Duration in the larval stage	6.26 ± 0.13 a	5.62 ± 0.09 b	4.11 (252)	<0.001 (0.336, 0.954)
Duration in the pupal stage	9.27 ± 0.14 a	10.84 ± 0.1 b	8.417 (163)	<0.001 (−1.965, 1.191)
APOPs	11.12 ± 0.78 a	9.74 ± 0.35 a	1.74 (72)	0.086 (−2.969, −0.201)
TPOPs	27.68 ± 0.89 a	26.69 ± 0.41 a	0.783 (72)	0.436 (−2.56, 0.116)

Different lowercase letters indicate significant differences in the responses (*p* ≤ 0.05) among the distances based on the results of Student’s *t*-test.

**Table 4:** Population parameters of the two strains of *P. okadai*.

Parameter	Resistant Strain (Mean ± SE)	Susceptible Strain (Mean ± SEM)	t (df)	*p* (95% Confidence Interval)
Intrinsic rate of increase, *r* (day^−1^)	0.0448 ± 0.0093 a	0.090 ± 0.0039 b	38.85 (199)	<0.001 (−0.49, −0.45)
Finite rate of increase, *λ* (day^−1^)	1.0458 ± 0.0097 a	1.0943 ± 0.0042 b	37.82 (199)	<0.001 (−0.52, −0.48)
Net reproductive rate, *R*_0_ (offspring)	6.3260 ± 2.1712 a	32.1565 ± 4.1889 b	14.15 (199)	<0.001 (−27.7, −25.8)
Mean generation time, *T* (days)	41.205 ± 1.7490 a	38.5930 ± 0.5900 a	0.31 (199)	0.757 (2.396, 3.062)

Different lowercase letters indicate significant differences in the responses (*p* ≤ 0.05) among the distances based on the results of Student’s *t*-test.

## Data Availability

Data are contained within the article.
